# One-pot synthesis of fuel precursor from acetoin fermentation broth using ionic liquid-based salting-out extraction system

**DOI:** 10.1186/s13068-023-02344-w

**Published:** 2023-06-02

**Authors:** Hanxiao Zhang, Yan Li, Jing Zhuang, Jianying Dai, Zhi-Long Xiu, Chunshan Quan

**Affiliations:** 1 School of Bioengineering, Dalian University of Technology, Dalian, 116024 People’s Republic of China; 2 Key Laboratory of Biotechnology and Bioresources Utilization, Dalian Minzu University, Dalian, 116650 People’s Republic of China

**Keywords:** Fuel precursor, Salting-out extraction, Hydroxylammonium ionic liquids, Aldol condensation, Acetoin

## Abstract

**Background:**

The development of biofuels, especially liquid hydrocarbon fuels, has been widely concerned due to the depletion of fossil resources. In order to obtain fuel precursors, the reaction of C–C bond formation is usually carried out with biomass derived ketones/aldehydes as reactants. Acetoin and 2,3-butanediol are two platform chemicals, which are co-existed in fermentation broth and traditionally separated by distillation, and then acetoin could be use as C4 building block to prepare hydrocarbon fuels. In order to mitigate the process complexity, direct aldol condensation reaction of acetoin in fermentation broth was studied in this work.

**Results:**

A one-pot process of product separation and acetoin derivative synthesis was proposed based on salting-out extraction (SOE). Aldol condensation reaction of acetoin and 5-methyl furfural in different SOE systems was compared, and the results showed that the synthesis of C_10_ fuel precursors and separation of C_10_ products and 2,3-butanediol from fermentation broth were achieved in one-pot with ethanolammonium butyrate (EOAB) and K_2_HPO_4_ as SOE reagents and catalysts. The SOE and reaction conditions such as the concentrations of EOAB and K_2_HPO_4_, reaction temperature and time were optimized. When the system was composed of 6 wt% EOAB-44 wt% K_2_HPO_4_ and the mixture was stirred for 6 h at 200 rpm, 40 ℃, the yield of C_10_ products was 80.7%, and 95.5% 2,3-butanediol was distributed to the top EOAB-rich phase. The exploration of reaction mechanism showed that an imine intermediate was rapidly formed and the subsequent C_10_ product formation was the key step for aldol condensation reaction.

**Conclusions:**

With EOAB and K_2_HPO_4_ as SOE reagents and catalysts, one-pot synthesis of fuel precursor from acetoin fermentation broth was achieved without prior purification. A yield of 80.7% for C_10_ products was obtained which was accumulated at the interface of two aqueous-phase, and 95.5% 2,3-BD was distributed to the top EOAB-rich phase. This work provides a new integration process of product separation and derivative synthesis from fermentation broth based on ionic liquid SOE.

**Graphical Abstract:**

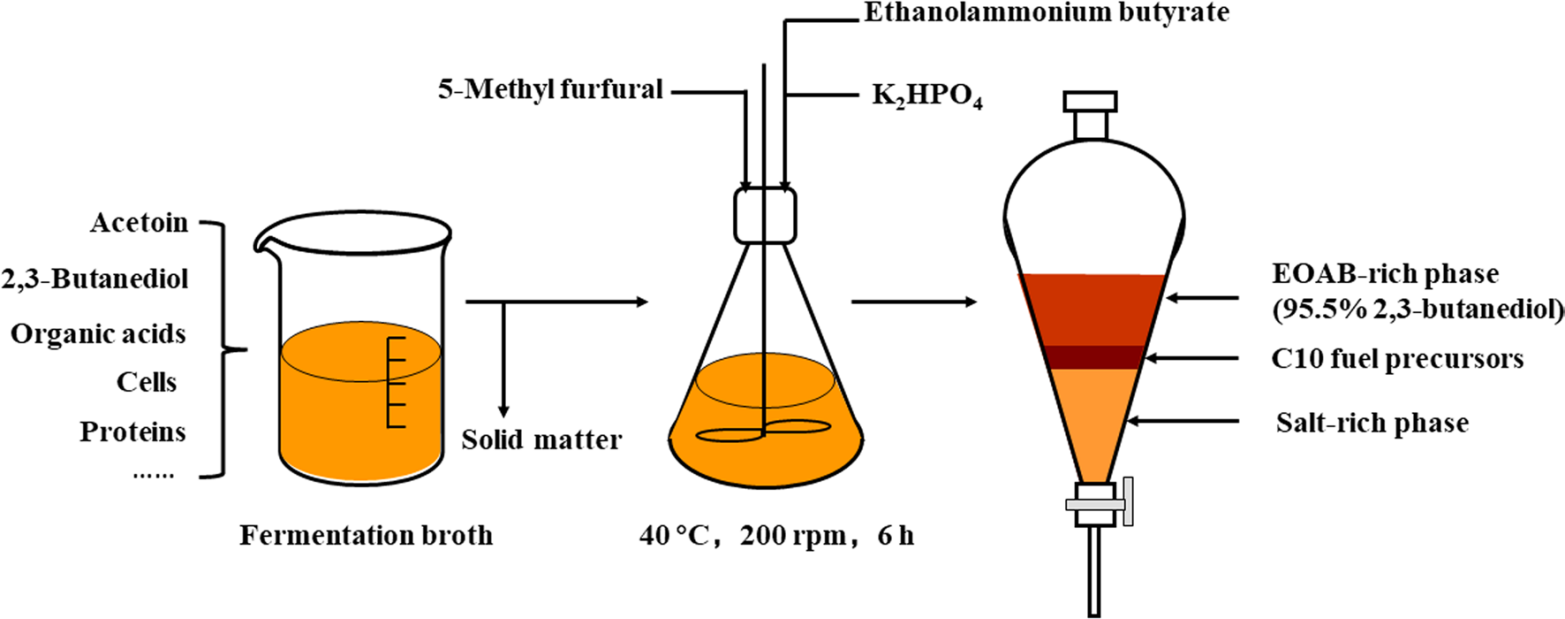

**Supplementary Information:**

The online version contains supplementary material available at 10.1186/s13068-023-02344-w.

## Background

Production of biofuels from renewable biomass is a sustainable and carbon-neutral way to replace fossil fuels gradually and reduce greenhouse gas emissions [1–3]. A variety of platform compounds (furan, furfural, and ketone) and biofuels such as oxygenated fuels (methanol, ethanol, and butanol) and hydrocarbon fuels (alkane, olefin, and arene) can be obtained from biomass via biotransformation or combination of chemical and biochemical conversion [4–6]. Generally, high-quality liquid fuels are saturated hydrocarbons with a large number of carbon atoms. However, the fermentation products such as ketones and alcohols are usually short-chain compounds. Therefore, the reactions such as aldol condensation reaction, alkylation reaction, Michael reaction, Guerbet reaction, and Diels–Alder reaction have been carried out to elongate the carbon chain [1]. Among them, much attention has been paid to the aldol condensation reaction of short-chain ketones and biomass derived carbon units [7]. For example, the precursors of liquid fuels were obtained from the reaction of furfural and ketone [8, 9].

Acetoin is one of promising sugar-derived platform chemicals [10], which can react with a variety of compounds to produce fuel precursors with long chain carbon [3]. In the natural metabolic pathways of microbes, acetoin is the precursor of 2,3-butanediol (2,3-BD), and both of them have been detected in fermentation broth while the concentration relied on the characteristics of the strain. 2,3-BD could be used as fuel [11], or converted to methyl ethyl ketone which could be used as a fuel additive and converted to C9/C14 fuel precursors by carbon chain extension [4, 12–14]. Therefore, both of them have to be recovered from fermentation broth.

In traditional way, pure compounds are used as raw materials to produce the derivative products [3, 4]. Thus, three main separation steps, including solid–liquid separation (centrifugation, membrane filtration, etc.), primary recovery, and final purification, are carried out to obtain pure product from the fermentation broth [15]. Acetoin and 2,3-BD are commonly separated during the distillation process due to their similar polarity but different boiling points[16]. However, a lot of energy is consumed in distillation [17]. In order to mitigate the process complexity and energy consumption, integration of product separation and derivative formation have been explored recently. For example, the coupling of butanol extraction from ABE fermentation broth with esterification process not only reduced product inhibition, but also improved butanol production [18, 19].

Salting-out extraction (SOE) is a convenient and low-cost way for the primary separation of bio-based chemicals from fermentation broths [20]. By salting-out extraction, acetoin and 2,3-BD were distributed to the top phase (solvent-rich), and most of the cells, proteins, by-products, and residual sugar could be removed. In recent years, ionic liquids (ILs) have been used as solvent in SOE, and part of ILs showed high efficiency in the separation of bio-based chemicals [21]. For example, hydroxylammonium ILs could form aqueous two-phase with K_3_PO_4_, and 92.7% of acetoin and 95.0% of 2,3-BD were recovered from the fermentation broth using the system of ethanolammonium butyrate (EOAB) and K_3_PO_4_ [22]. In addition, ionic liquids are efficient catalysts in some chemical reactions [23]. For example, ethanolammonium acetate (EOAA) could catalyze the aldol condensation reaction of acetoin and aldehydes derived from lignocellulose to obtain fuel precursors, and the yield of 87% was obtained from the reaction between acetoin and 5-methyl furfural (5-MF) [3].

Encouraged by the application of EOAB in the SOE and EOAA in the aldol condensation reaction [3, 22], the supernatant of fermentation broth was tried in the aldol condensation reaction of acetoin and 5-MF, and the integration of 2,3-BD separation and acetoin reaction based on EOAB and salt was explored in this work. A one-pot method of product separation and derivative synthesis was developed. After reaction, acetoin in fermentation broth was converted to C_10_ fuel precursors which were accumulated in the middle phase, and 2,3-BD was partitioned into the top EOAB-rich phase, thus the derivative formation and product separation were achieved in one-pot.

## Materials and methods

### Materials

Acetoin (95%, LOT: J1311040), 2,3-BD (98%, LOT: K1904182) and 5-MF (98%, LOT: C2020131) were purchased from Aladdin Reagent Co., Ltd. (China). Anhydrous K_2_HPO_4_ was purchased from MacLean Reagent Co., Ltd. (China). Ethanolamine (99%, LOT: 20210304) and butyric acid (99%, LOT: 20201028) were purchased from Sinopharm Chemical Reagent Co., Ltd (China), and the ionic liquid EOAB was synthesized by the acid–base neutralization method [24].

### Preparation of fermentation broth

The fermentation broth was prepared by fed-batch fermentation with industrial glucose as a carbon source. *Bacillus subtilis* (CGMCC No. 23179), a mutant strain of *B. subtilis* DL01 which was isolated from sea sediment [25], was used for fermentation. The seed medium was composed of 40 g/L glucose, 1.5 g/L yeast extract, 6 g/L (NH_4_)_2_HPO_4_, 8 g/L KH_2_PO_4_, and 0.1 g/L MnSO4 (pH7.0), and the fermentation medium was composed of 140 g/L glucose, 7 g/L yeast extract, 12 g/L corn steep liquor powder, 6 g/L KH_2_PO_4_, and 0.13 g/L MnSO_4_. The fed-batch fermentation was carried out in a 5-L stirred bioreactor (BIOTEC-5JG-7000A, BXBIO, China) with working volume of 2 L. The preserved strain was grown for 12 h at 37 ℃, 200 rpm in the LB medium, and then transferred to the seed medium (2%, v/v) and cultured for 14 h at 37 ℃, 200 rpm. The obtained seed was inoculated (10%, v/v) into the fermentation medium. The fermentation was carried out for 80 h at 37 ℃ under the oxygen supply condition of 0.25 vvm, 350 rpm. Glucose and (NH_4_)_2_HPO_4_ (mass ratio = 10:1) was supplemented when residual glucose in fermentation broth was less than 20 g/L. During the fermentation process, the pH value was not adjusted by automatic addition of 5 mol/L NaOH unless the pH value was less than 5.7. The fermentation broth was centrifuged at 5000 rpm for 30 min. The supernatant was stored at – 20 ℃ for the following SOE and reaction experiments. The concentrations of components in the supernatant were as follows: acetoin 65 g/L, 2,3-BD 40 g/L, citric acid 10 g/L and lactic acid 5 g/L.

An aqueous solution containing 65 g/L acetoin and 40 g/L 2,3-BD was prepared for the following experiment, which was also used as the control to check the effect of the impurities in fermentation broth on aldol condensation reaction.

### Effect of salt on the reaction of acetoin and 5-methyl furfural

The reaction was carried out for 18 h at 37 ℃ and 200 rpm in a shaking incubator. Aqueous solution of acetoin and 2,3-BD 10 mL, 0.04 mol salt, and 1.1 g EOAB were added into a 50-mL conical flask and vortexed for dissolution. Then, 5-MF was added to an equal mole of acetoin for reaction. After reaction, the system was divided into three phases, in which EOAB and 2,3-BD were enriched in the top phase, C_10_ fuel precursors was accumulated in the middle phase, and the salt was rich in the bottom phase. The top phase was taken out for acetoin and 2,3-BD determination, and then ethyl acetate was added to extract C_10_ product and residual 5-MF for GC analysis. The conversion of 5-MF (*α*_MF_), and the selectivity (*S*_C10_) and yield (*Y*_C10_) of C_10_ products were defined as follows:

1 $$\alpha_{{{\text{MF}}}} = {\text{ Mole of consumed 5}} - {\text{MF}}/{\text{mole of initial 5}} - {\text{MF,}}$$

2 $$S_{{{\text{C1}}0}} = {\text{ Total peak area of C1}}0{\text{ products}}/{\text{total peak area of all the products,}}$$

3 $$Y_{{{\text{C1}}0}} = \alpha_{{{\text{MF}}}} \times S_{{{\text{C1}}0}} .$$

### Salting-out extraction of acetoin and 2,3-butanediol

To obtain an appropriate SOE condition for the coupled aldol condensation reaction, the effect of different concentrations of K_2_HPO_4_ and EOAB on the separation of acetoin and 2,3-BD from fermentation broth was investigated. The fermentation broth and K_2_HPO_4_ were added into a 25-mL tube and vortexed until the salt dissolved, and then EOAB was added and vortexed for 1 min. The total weight of the system was 10 g, and the final concentration of K_2_HPO_4_ in the system was 35 wt%, 41 wt%, and 47 wt% while the final concentration of EOAB was 3 wt%, 6 wt%, and 8 wt%, respectively. The mixture stood at room temperature for 2 h. The concentrations of acetoin, 2,3-BD and EOAB in the top phase were analyzed. The partition coefficient (*K*) and recovery (*Y*) were defined as follows:

4 $$K_{{\text{j}}} = C_{{{\text{jt}}}} /C_{{{\text{jb}}}} ,$$

5 $$Y_{{\text{j}}} = V{\text{t}} \times C_{{{\text{jt}}}} /\left( {V_{0} \times C_{{{\text{j}}0}} } \right),$$

where *V*_t_ and *V*_0_ represented the volume of top phase and fermentation broth added, respectively; *C*_jt_, *C*_jb_ and *C*_j0_ represented the concentration of chemical j in the top phase, bottom phase, and fermentation broth, respectively.

### Optimization of the reaction conditions of acetoin and 5-methyl furfural

The reaction was carried out in a magnetic stirring water bath. The temperature and stirring speed varied from 40 ℃ to 60 ℃, and 200 rpm to 600 rpm, respectively. The fermentation broth and K_2_HPO_4_ were added into a 50-mL conical flask and stirred for dissolution. Then, EOAB and 5-MF were added. The total weight of fermentation broth, K_2_HPO_4_ and EOAB was 20 g. The mole ratio of 5-MF to acetoin was 1:1.

### Analytical methods

The concentrations of fuel precursors, 5-MF, acetoin, and 2,3-BD were analyzed by gas chromatograph (GC-2010, SHIMADZU, Japan) equipped with a capillary column and FID detector. The concentrations of acetoin and 2,3-BD were analyzed by a chiral capillary column BGB-174 (30 m × 0.25 mm × 0.25 μm), while 5-MF and fuel precursors were analyzed by a capillary column HP-5MS (30 m × 0.25 mm × 0.25 μm).

The concentrations of EOAB, organic acids, and residual glucose in the fermentation broth were analyzed by HPLC equipped with an Aminex HPX-87H column (300 × 7.8 mm) and a refractive index detector (Waters 2414), and 5 mmol/L sulfuric acid was used as mobile phase at a flow rate of 0.6 mL/min.

### Statistics

Each experiment was carried out in triplicate. The mean experimental values with standard deviations are given in the tables and figures.

## Results and discussion

### EOAB-based SOE of acetoin and 2,3-butanediol from fermentation broths

Our previous work showed that hydroxylammonium ionic liquids could form aqueous two-phase with tripotassium orthophosphate (K_3_PO_4_), while phase split was not observed with saturated aqueous solution of dipotassium hydrogenphosphate (K_2_HPO_4_), sodium carbonate (Na_2_CO_3_), ammonium sulfate ((NH_4_)_2_SO_4_), and sodium acetate (CH_3_COONa) at 25 ℃ due to the high hydrophilicity of ILs [22]. On the other hand, it has been found that acetoin and 2,3-BD took part in the phase split during SOE, and less amount of solvent for phase split was required under the existence of acetoin or 2,3-BD [20, 21, 26]. In addition, inorganic salts showed catalytic activity in many studies. For example, potassium carbonate (K_2_CO_3_) could catalyze aldol condensation reaction [27–30], while K_3_PO_4_, K_2_HPO_4_ and K_2_CO_3_ were effective catalysts for nucleophilic addition reaction [31, 32]. Considering that aldol condensation reaction was carried out via alkaline catalytic mechanism when EOAA was used as catalysts [3], five alkaline salts (K_3_PO_4_, K_2_HPO_4_, K_2_CO_3_, potassium pyrophosphate (K_4_P_2_O_7_), and potassium acetate (CH_3_COOK)) were selected for SOE and reaction, since alkaline salts could remove the α-H of the carbonyl group to form a carbon anion in nucleophilic addition reaction.

As shown in Table 1, EOAB could form aqueous two-phase with fermentation broth containing 4 mol/L K_3_PO_4_, K_2_HPO_4_, K_2_CO_3_, or K_4_P_2_O_7_, in which most of acetoin, 2,3-BD and EOAB were partitioned to the top phase. Since the products of C_10_ fuel precursors were insoluble in water, three phases were observed after reaction except CH_3_COOK. The products accumulated at the interphase between the two aqueous phases, in which the top phase was rich in EOAB and 2,3-BD while the bottom phase was rich in salt, thus the recovery of 2,3-BD from fermentation broth and synthesis of acetoin derivative were achieved in one-pot.

**Table 1:** Results of the reaction between acetoin and 5-MF in different SOE systems

	System		Conversion of 5-MF (%)	C10 products		Phase split	
				Selectivity (%)	Yield (%)	Mixture without 5-MF	After reaction*
1	EOAB	–	23.2 ± 0.3	98.9 ± 1.0	23.1 ± 0.5	H	TLP^b^
2	–	K_3_PO_4_	84.3 ± 1.2	51.2 ± 0.1	42.8 ± 0.1	AT	TLP^a^
3	EOAB	K_3_PO_4_	98.4 ± 0.3	9.0 ± 0.5	8.9 ± 0.6	AT	TRP
4	–	K_2_CO_3_	73.7 ± 1.3	55.9 ± 0.1	41.3 ± 0.8	AT	TLP^a^
5	EOAB	K_2_CO_3_	88.2 ± 0.1	7.3 ± 0.3	6.4 ± 0.3	AT	TRP
6	–	K_4_P_2_O_7_	23.2 ± 2.1	94.6 ± 0.2	22.0 ± 2.0	AT	TLP^a^
7	EOAB	K_4_P_2_O_7_	93.5 ± 0.3	48.6 ± 1.2	45.4 ± 0.4	AT	TRP
8	–	K_2_HPO_4_	6.53 ± 0.0	93.2 ± 0.1	6.08 ± 0.01	AT	TLP^a^
9	EOAB	K_2_HPO_4_	97.9 ± 0.1	67.1 ± 0.2	65.8 ± 0.1	AT	TRP
10	–	CH_3_COOK	7.56 ± 0.8	0	0	H	H
11	EOAB	CH_3_COOK	63.7 ± 2.4	81.1 ± 0.4	55.2 ± 1.6	H	TLP^b^

^*^The reaction system was composed of 10 mL aqueous solution of acetoin and 2,3-BD, 0.04 mol salt and/without 1.1 g EOAB, and carried out at 37 °C and 200 rpm for 18 h

*H* homogeneous, *AT* aqueous two-phase, *TLP*^a^ two liquid phases where the top liquid phase was the C_10_ products, *TLP*^b^ two liquid phases where the bottom liquid phase was the C_10_ products, *TRP* three phases, where the liquid of C_10_ products accumulated at the interface of two aqueous phases and the top phase was rich in EOAB and 2,3-BD

### Aldol condensation reaction of acetoin and 5-methyl furfural in different SOE systems

**Fig. 1:**
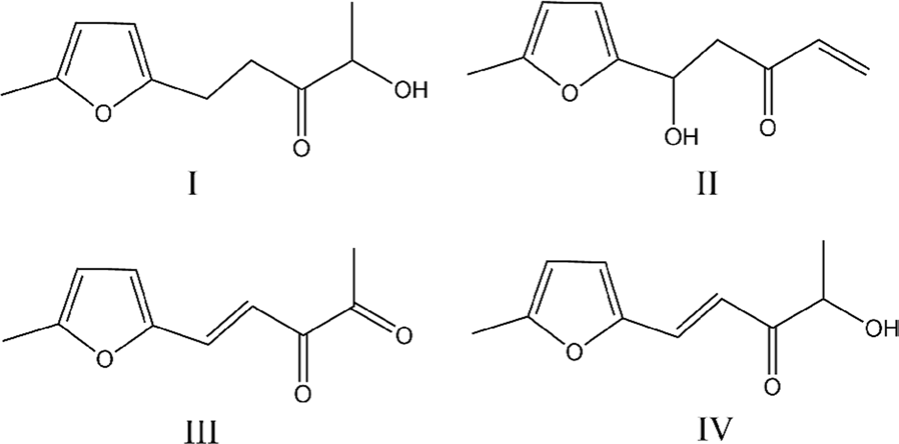
Structures of the C_10_ products identified in the reaction of acetoin and 5-MF catalyzed by EOAB and alkaline salts

As shown in Fig. 1, four C_10_ products were identified in the aldol condensation reaction of acetoin and 5-MF, in which compound IV was the main product and reported before [3]. All these C_10_ compounds could be converted to hydrocarbon fuel by hydrodeoxygenation, so the yield of C_10_ products was the sum of the four compounds (GC–MS and NMR are shown in Additional file 1).

Different catalytic efficiency was observed in the reaction of acetoin and 5-MF when salt was existed. As shown in Table 1, the selectivity of C_10_ products (*S*_C10_) was about 99% when only 10% EOAB was used as catalyst, while the conversion of 5-MF (*α*_MF_) was only 23%. When only the salt K_3_PO_4_ or K_2_CO_3_ was used as catalysts, *α*_MF_ was greater than 70% while *S*_C10_ was less than 60%, probably due to the steric effect of reactants. For example, in the reaction of formaldehyde and propionaldehyde catalyzed by K_2_CO_3_, a yield of 67.8% was obtained [33]. When K_4_P_2_O_7_ or K_2_HPO_4_ was used as catalyst, *S*_C10_ was greater than 90%, while *α*_MF_ was less than 30%, resulting in a low yield of C_10_ products (*Y*_C10_).

The synergistic effect of catalysis could be observed when both salt and EOAB were existed. The value of *α*_MF_ was significantly increased, while *S*_C10_ was decreased, except CH_3_COOK where both *α*_MF_ and *S*_C10_ were increased. The *S*_C10_ obtained from the systems of EOAB–K_3_PO_4_ and EOAB–K_2_CO_3_ decreased to less than 10%, and the main product became an imine intermediate, which was identified in the later experiment, thus the yield went down greatly. Among these systems, the combination of K_2_HPO_4_ and EOAB demonstrated high *α*_MF_ and medium *S*_C10_, so the highest *Y*_C10_ of 66% was obtained under present condition. The concentration of K_2_HPO_4_ in this reaction was near to saturation, so it was possible to improve *S*_C10_ by reducing the amount of K_2_HPO_4_ in the system.

### ***Simultaneous aldol condensation and product separation in SOE system of EOAB and K***_***2***_***HPO***_***4***_

**Fig. 2:**
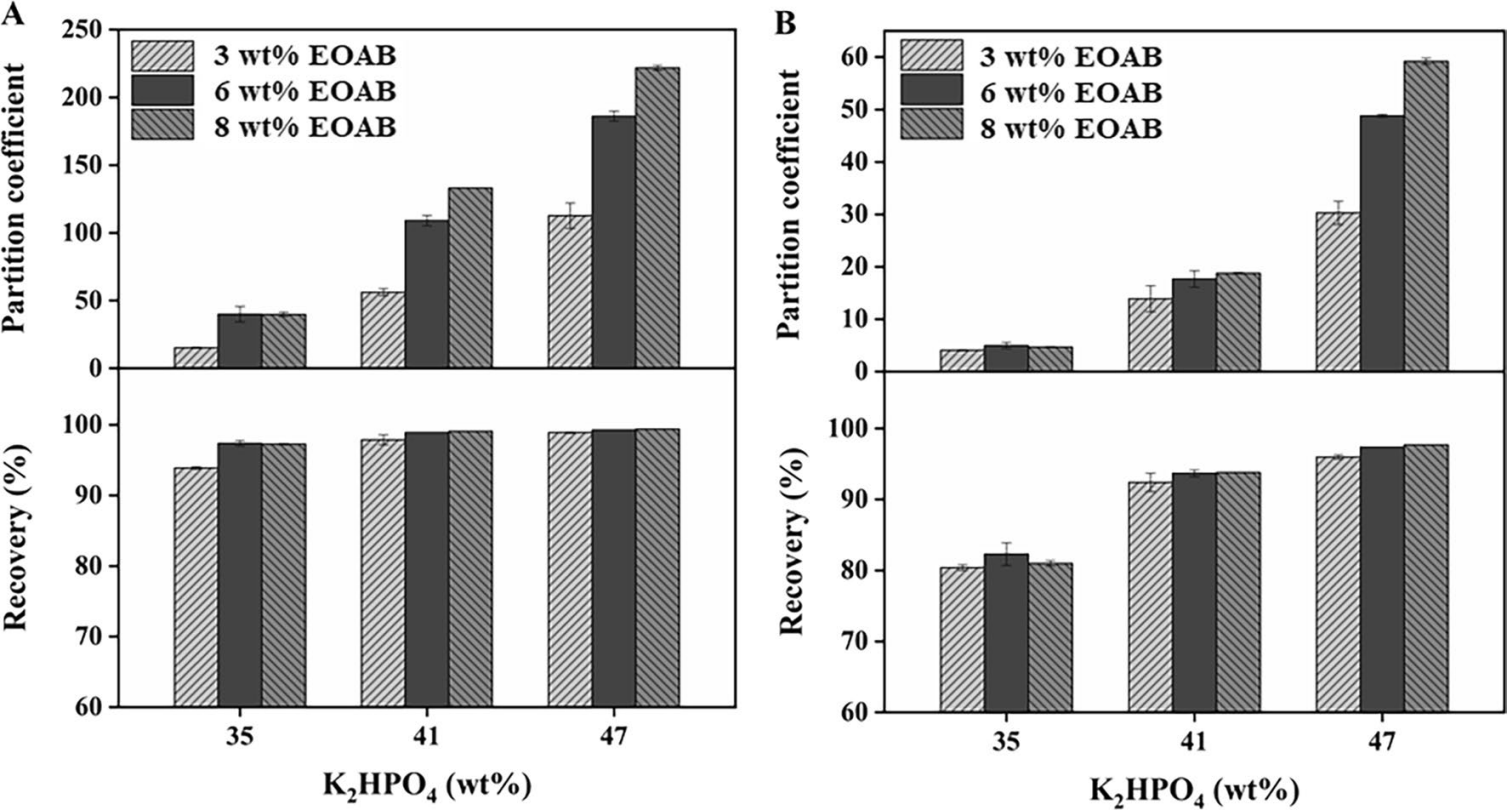
Partition behavior of acetoin and 2,3-BD in EOAB–K_2_HPO_4_ system. **A** acetoin; **B** 2,3-BD

The SOE of acetoin and 2,3-BD from fermentation broth was explored at different concentrations of EOAB and K_2_HPO_4_. As shown in Fig. 2, the partition coefficient and recovery of acetoin and 2,3-BD increased with the increasing concentration of EOAB and K_2_HPO_4_. Under the same condition, the partition coefficient and recovery of 2,3-BD were lower than that of acetoin, respectively, which was related to their structure and the extraction mechanism of EOAB [22]. The salt demonstrated a great influence on the distribution of acetoin and 2,3-BD when EOAB was at a low concentration of 3 ~ 8 wt%. When K_2_HPO_4_ concentration was greater than 41 wt%, the 2,3-BD recovery was higher than 90% while acetoin recovery was higher than 95%. Under the condition of 8 wt% EOAB-47 wt% K_2_HPO_4_, the highest partition coefficients of acetoin and 2,3-BD were obtained, which were 221.8 and 59.2, respectively.

In this work, EOAB and K_2_HPO_4_ not only acted as extractant and salting-out reagent but also catalysts, so the effect of their concentrations on the aldol condensation reaction was also determined. Generally, the composition of fermentation broth is very complex, including organic acids, residual sugars and proteins, which might affect the catalytic efficiency. Therefore, pure chemicals were used in the derivatization of bio-based chemicals to prepare fuels while few studies were reported to use fermentation broth directly [3, 34, 35]. As shown in Table 2, there was no significant difference in* S*_C10_, while *α*_MF_ from the fermentation broth was a little lower than that from the aqueous solution, thus a slightly decreased *Y*_C10_ was obtained. This result indicated that it was feasible to use fermentation broth for aldol condensation reaction without prior purification.

**Table 2:** Reaction of acetoin and 5-MF in EOAB–K_2_HPO_4_ system

Solution	System		Conversion of 5-MF (%)	Products of C10	
	EOAB (wt%)	K_2_HPO_4_ (wt%)		Selectivity (%)	Yield (%)
Aqueous solution	6	41	98.3 ± 0.1	81.0 ± 0.7	79.6 ± 0.7
Fermentation broth	6	41	96.3 ± 0.2	78.6 ± 1.2	75.6 ± 1.0
	6	44	98.0 ± 0.1	77.5 ± 1.4	76.0 ± 1.4
	3	47	82.0 ± 0.7	85.1 ± 1.1	69.8 ± 0.3
	6	47	98.8 ± 0.1	74.7 ± 0.1	73.8 ± 0.1
	8	47	99.4 ± 0.1	71.6 ± 1.2	71.2 ± 1.2
	10	47	99.6 ± 0.1	69.2 ± 0.4	68.9 ± 0.5

The influences of the concentrations of EOAB and K_2_HPO_4_ on the aldol condensation reaction are shown in Table 2. At K_2_HPO_4_ concentration of 47 wt%, significant improvement of *α*_MF_ was observed when EOAB concentration was increased from 3 wt% to 6 wt%, while *S*_C10_ decreased with increased EOAB concentration from 3 wt% to 10 wt%. Under the condition of 6 wt% EOAB, both *α*_MF_ and *S*_C10_ changed little with the increasing concentration of K_2_HPO_4_. These results showed that high concentration of EOAB and K_2_HPO_4_ was not beneficial for the *S*_C10_ improvement, resulting in the increase of by-products.

Under the condition of 6 wt% EOAB, the highest *Y*_C10_ of 76.0% was obtained in range of 41 ~ 44 wt% K_2_HPO_4_. However, 2,3-BD recovery at 44 wt% K_2_HPO_4_ was 95.5%, higher than that at 41 wt% K_2_HPO_4_. Therefore, the system 6 wt% EOAB–44 wt% K_2_HPO_4_ was finally selected for the integration of reaction and 2,3-BD separation from the fermentation broth.

### Optimization of one-pot synthesis of fuel precursors

**Fig. 3:**
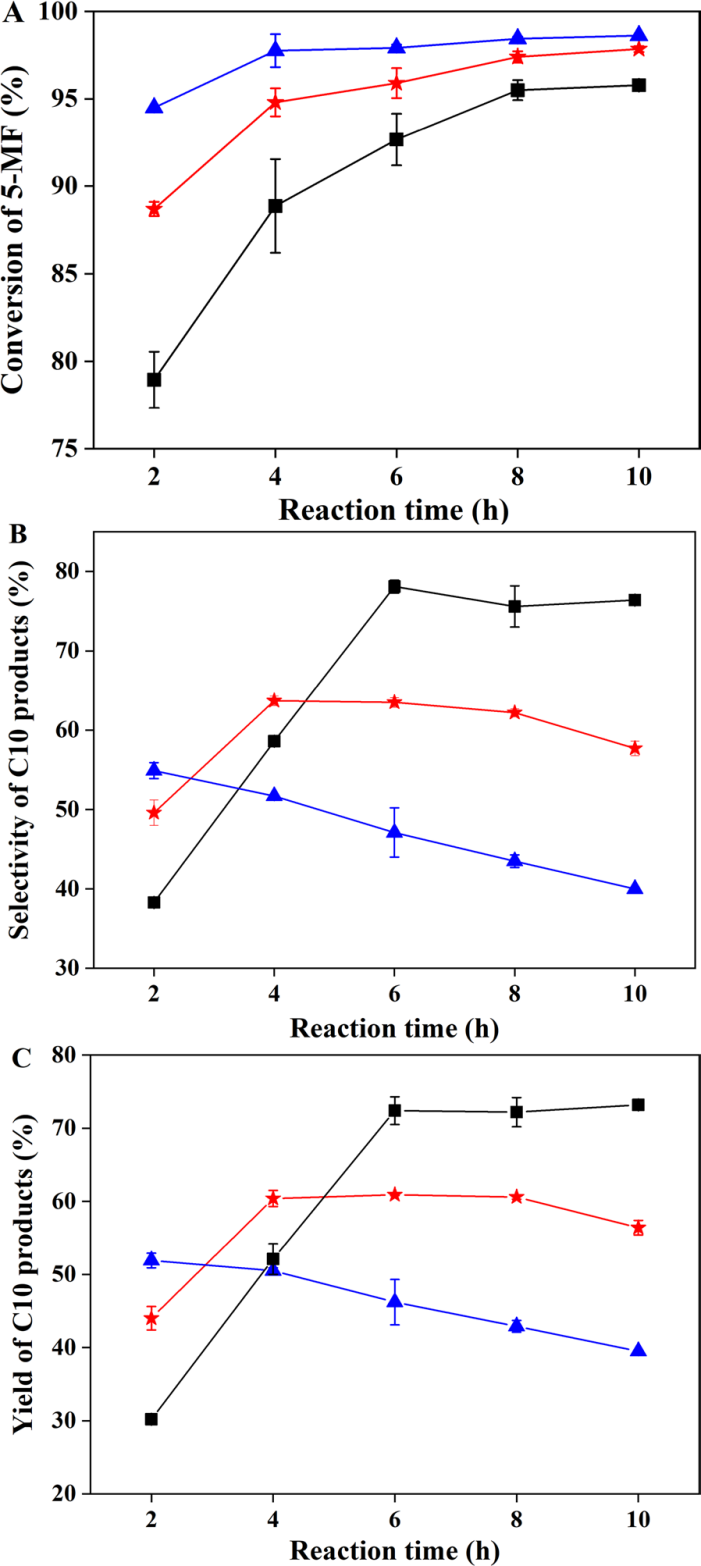
Time courses of the aldol condensation reaction of acetoin and 5-MF at different temperatures and 600 rpm. **A** conversion of 5-MF; **B** selectivity of C_10_ products; **C** yield of C_10_ products. square, 40 ℃; star, 50 ℃; triangle 60 ℃

Generally, the aldol condensation reaction under alkaline condition is mild. For example, the reaction catalyzed by amino acids and other organic small molecules was performed at room temperature [36, 37], while the reaction of acetoin with furfural, 5-hydroxyl furfural, or 5-MF was carried out 50 ℃ [3]. Therefore, the effect of temperature on the reaction was explored, and samples were taken every 2 h to monitor the reaction progress. As shown in Fig. 3, *α*_MF_ was improved with increase in temperature. The detection of residual 5-MF showed that at 60 ℃ *α*_MF_ reached the highest of 97.7% in 4 h, while at 40 ℃ and 50 ℃ the highest *α*_MF_ was obtained in 8 h. *S*_C10_ decreased linearly with time extension at 60 ℃. At 40 ℃ and 50 ℃, *S*_C10_ firstly increased and then decreased with time extension. The highest *S*_C10_ and *Y*_C10_ were obtained at 40 ℃ after 6 h reaction.

**Fig. 4:**
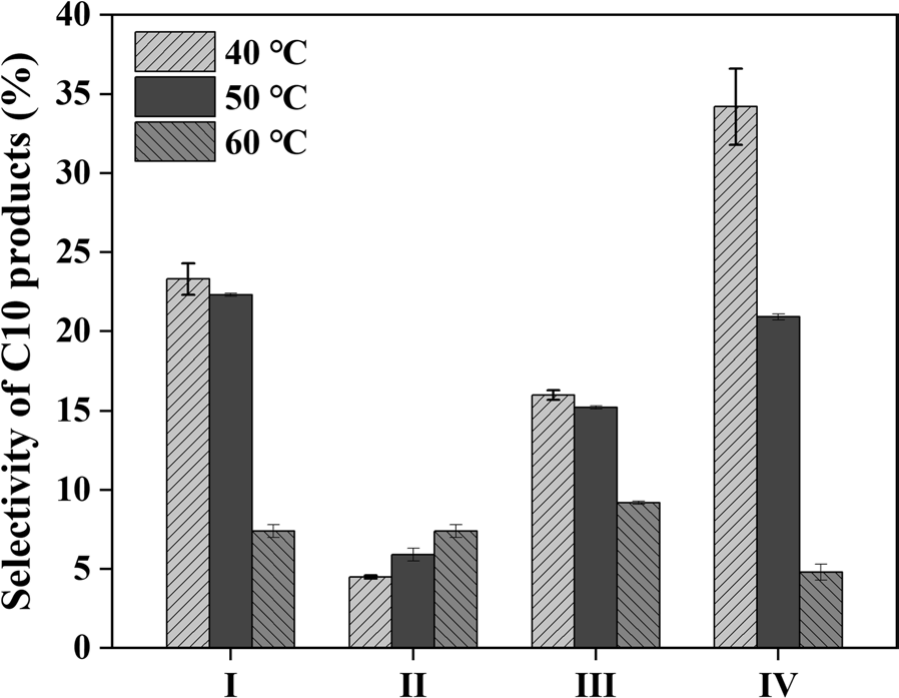
Effect of temperature on the selectivity of four C_10_ products after 10 h reaction under 600 rpm

As shown in Fig. 4, the selectivity of four C_10_ products was influenced by temperature. With temperature increasing, the selectivity of product I, III and IV decreased while the selectivity of product II increased. Typically, the selectivity of product IV decreased 29.4% when temperature increased from 40 ℃ to 60 ℃. Thus, higher temperature made the C_10_ products react further and more by-products were generated.

To avoid the deviation of results caused by uneven sampling and long sampling intervals, the points with high yield at different temperatures (Fig. 3C) were repeated, and the effect of agitation was also compared. As shown in Table 3, under the stirring speed of 600 rpm, *Y*_C10_ at 40 ℃ and 45 ℃ was significantly higher than that at 50 ℃ and 60 ℃, and there was no significant difference between the *Y*_C10_ at 40 ℃ and 45 ℃. Therefore, the influence of stirring speed on the reaction was explored under these two temperatures. Theoretically, high stirring speed could promote the mixing of substances in the system and facilitate the reaction because 5-MF is insoluble in water. In fact, there was no significant difference in *α*_MF_, *S*_C10_ and *Y*_C10_ in the range of 200 rpm–600 rpm, indicating that the stirring speed of 200 rpm could provide sufficient mixing efficiency for the reaction. Therefore, the reaction condition of 40 ℃ and 200 rpm for 6 h was selected, and the yield of C_10_ products was 80.7%. Compared with the yield of 87% using pure acetoin and EOAA catalyst [3], the yield was a little lower in this work. However, the supernatant of fermentation broth was used directly for reaction without purification, the reaction temperature was 10 ℃ lower, and another product 2,3-BD was also separated. Therefore, the one-pot method is worth further study in the derivative synthesis from bio-based chemicals in fermentation broth.

**Table 3:** Results of the reaction carried at different agitation, temperatures, and time

T (°C)	t (h)	Stirring speed (rpm)	Conversion of 5-MF (%)	Products of C_10_	
				Selectivity (%)	Yield (%)
60	0.5	600	80.8 ± 0.1	69.0 ± 1.0	55.8 ± 0.9
	1		87.8 ± 1.9	66.5 ± 1.7	58.4 ± 2.8
	2		95.8 ± 0.5	63.9 ± 0.4	61.2 ± 0.6
50	3	600	91.2 ± 0.4	75.3 ± 0.6	68.6 ± 0.2
	4		96.4 ± 0.2	79.2 ± 0.4	76.4 ± 0.2
	5		97.1 ± 0.6	78.0 ± 0.7	75.8 ± 1.1
45	6	600	97.0 ± 0.1	82.0 ± 0.4	79.5 ± 0.5
		400	96.8 ± 0.4	80.4 ± 1.9	77.8 ± 2.2
		200	96.4 ± 0.3	80.7 ± 0	77.7 ± 0.3
40	6	600	95.3 ± 0.3	82.3 ± 1.0	78.4 ± 0.7
		400	95.4 ± 0.3	84.2 ± 1.3	80.3 ± 1.5
		200	94.3 ± 0.3	85.0 ± 1.1	80.7 ± 0.8

### Mechanism of aldol condensation reaction

**Fig. 5:**
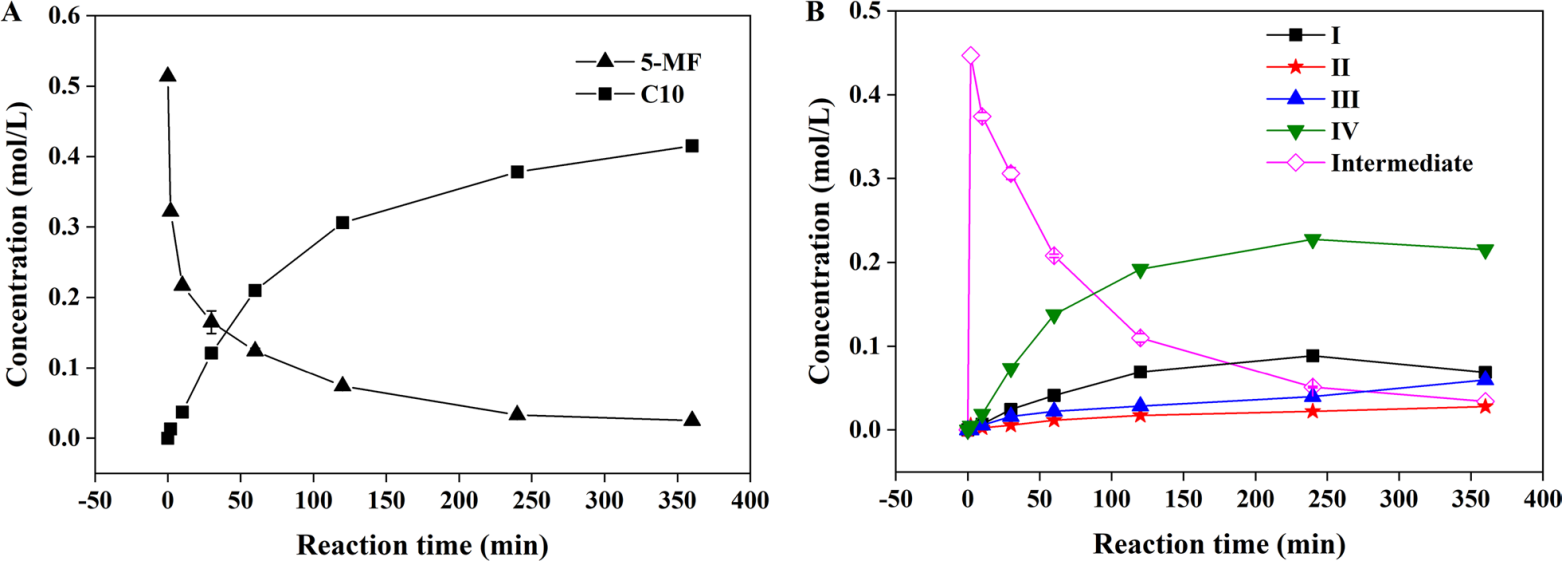
Time courses of the aldol condensation reaction of acetoin and 5-MF. Reaction conditions: 6 wt% EOAB-44 wt% K_2_HPO_4_; n(AC):n(5-MF) = 1:1; 40 ℃, 200 rpm. **A** 5-MF consumption and formation of C_10_ products; **B** formation of the intermediate and four C_10_ products

To explore the mechanism of co-catalysis of EOAB and K_2_HPO_4_, the time courses of 5-MF consumption and formation of products at 40 ℃ were monitored. As shown in Fig. 5A, 5-MF was rapidly consumed within 10 min and then the rate gradually slowed down. However, the total concentration of C_10_ products increased slowly, indicating a slow formation rate compared with 5-MF consumption. Similar phenomenon has been reported in the study of formaldehyde and propionaldehyde catalyzed by hydroxylammonium ionic liquid [38].

**Fig. 6:**
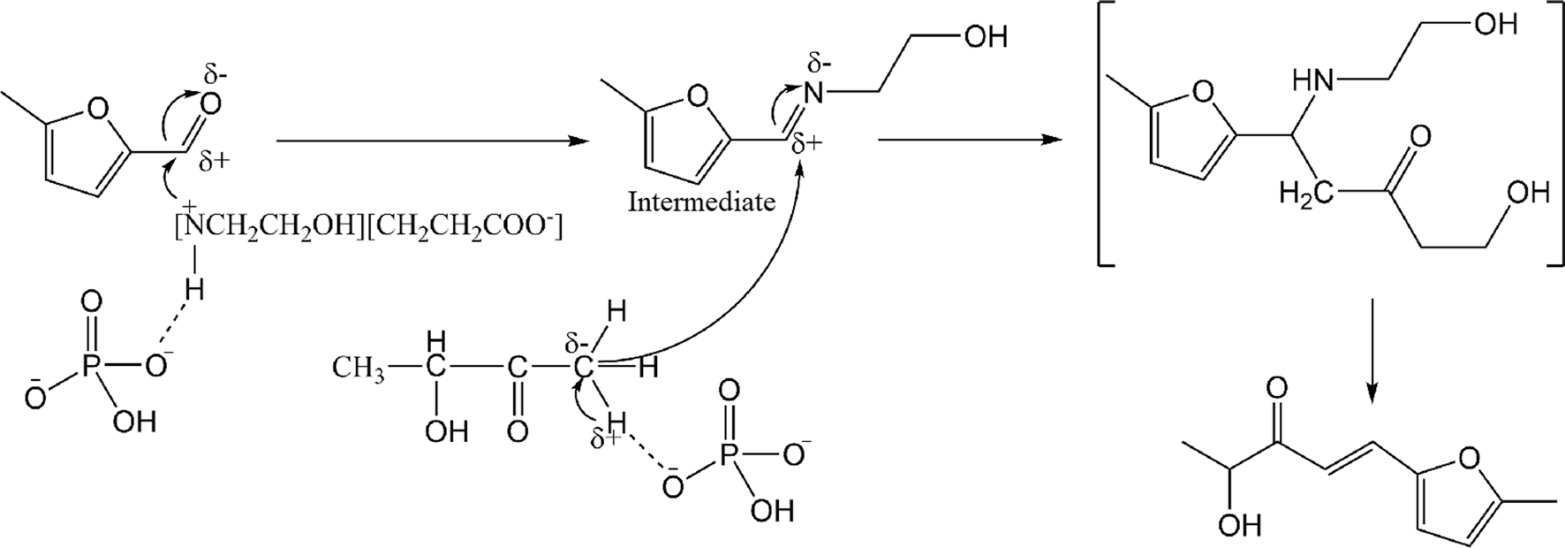
Tentative mechanism for the reaction of acetoin and 5-MF under the existence of EOAB and K_2_HPO_4_

The time courses of the four products formation are shown in Fig. 5B. With the prolongation of the reaction time, the concentrations of the four products gradually increased, but the formation rates of the four products were significantly different. The product of I and the product of IV were detected in 2 min while II and III were detected at 5 min, and the concentration of product IV was the highest. In addition, an intermediate was detected, which was rapidly formed and reached its highest concentration within 2 min, and then gradually decreased with the elongation of reaction time. The intermediate was identified as an imine by GC–MS and ^1^H NMR (structure shown in Fig. 6).

When a catalyst containing amino group was used in the aldol condensation reaction, it was found that an intermediate of imine was firstly formed, and then converted to aldol condensation product. For example, in the aldol condensation of propionaldehyde and formaldehyde, imine intermediates were detected when diethylammonium acetate or proline was used as catalyst [38, 39]. The formation of imine was more efficient and milder than that of direct condensation [40], and the rate-determining step in the reaction was the formation of C–C bond [41]. Based on the above analysis, it could be concluded that 5-MF firstly formed an imine intermediate with EOAB in this work, and then combined with acetoin to generate C_10_ products. The reaction between the intermediate and acetoin was the rate-limiting step. A tentative mechanism is depicted in Fig. 6. The HPO_4_^−^ anion could form a hydrogen-bonding net with EOAB and acetoin, respectively, which facilitate the dissociation of amine ion to react with 5-MF, and remove the *α*-H of the carbonyl group of acetoin to react with the intermediate. The accumulation of imine intermediate was also observed in the reaction catalyzed by EOAB and K_3_PO_4_/K_2_CO_3_. The main reason was that the alkaline environment in EOAB–K_3_PO_4_/K_2_CO_3_ system made the imine intermediate stable and unable to convert to C_10_ products.

## Conclusions

The integration of the synthesis of C_10_ fuel precursors and the separation of C_10_ products and 2,3-BD from fermentation broth was established by using EOAB and K_2_HPO_4_ as catalysts and SOE reagents. When the system contained 6 wt% EOAB and 44 wt% K_2_HPO_4_ and mixed for 6 h at 40 ℃, 200 rpm, the conversion of 5-MF reached 94.3%, the selectivity of C_10_ products was 85.0% and the yield of C_10_ products was 80.7%. Meanwhile, 95.5% 2,3-BD in fermentation broth was distributed to the top EOAB-rich phase and the C_10_ products accumulated at the middle phase. During the aldol condensation reaction, an imine intermediate was formed firstly, and then followed the formation of C_10_ products which was the rate-limiting step.

## Supplementary Information

**Additional file 1 of one-pot synthesis of fuel precursor from acetoin fermentation broth using ionic liquid-based salting-out extraction system : Fig. S1** GC analysis of the aldol condensation reaction of acetoin and 5-MF. t_R_ 6.653 min, 5-MF; t_R_ 13.506 min, Product I; t_R_ 15.562 min, Product II; t_R_ 16.555 min, Product III; t_R_ 17.846 min, Product IV; t_R_ 14.687 min, imine intermediate. **Fig. S2** GC–MS diagram of the four C10 products and intermediate. **A** Product Ipent-1-en-3-one); **B** product IIpentan-3-one); **C** product IIIpent-4-ene-2,3-dione); **D** product IVpent-1-en-3-one); **E** intermediatemethylene)amino)ethan-1-ol).** Fig. S3** ^1^H NMR spectra of product IV synthesized from 5-MF and AC.** Fig. S4** ^1^H NMR spectra of intermediate.

## Data Availability

All relevant data are included in the article and/or its Additional files 1. The data sets used and/or analyzed during the current study are available from the corresponding authors on reasonable request.

## Abbreviations

SOE: Salting-out extraction
EOAB: Ethanolammonium butyrate
EOAA: Ethanolammonium acetate
2,3-BD: 2,3-Butanediol
5-MF: 5-Methyl furfural
ILs: Ionic liquids
