# Supplementary material for: One-pot synthesis of fuel precursor from acetoin fermentation broth using ionic liquid-based salting-out extraction system
Source: Biotechnol Biofuels Bioprod. 2023 Jun 2;16:94. doi: 10.1186/s13068-023-02344-w (PMC10236752; doi:10.1186/s13068-023-02344-w)
Supplement: Supplementary file 1 — Additional file 1 of one-pot synthesis of fuel precursor from acetoin fermentation broth using ionic liquid-based salting-out extraction system : Fig. S1 GC analysis of the aldol condensation reaction of acetoin and 5-MF. tR 6.653 min, 5-MF; tR 13.506 min, Product I; tR 15.562 min, Product II; tR 16.555 min, Product III; tR 17.846 min, Product IV; tR 14.687 min, imine intermediate. Fig. S2 GC–MS diagram of the four C10 products and intermediate. A Product Ipent-1-en-3-one); B product IIpentan-3-one); C product IIIpent-4-ene-2,3-dione); D product IVpent-1-en-3-one); E intermediatemethylene)amino)ethan-1-ol). Fig. S3 1H NMR spectra of product IV synthesized from 5-MF and AC. Fig. S4 1H NMR spectra of intermediate. [file 13068_2023_2344_MOESM1_ESM.docx]

**One-pot synthesis of fuel precursor from acetoin fermentation broth**

**using ionic liquid-based salting-out extraction system**

Hanxiao Zhang^1^ Yan Li^1^ Jing Zhuang^1^ Jianying Dai^1^* Zhi-Long Xiu^1^ Chunshan Quan^2^

^1^School of Bioengineering, Dalian University of Technology, Dalian 116024, P. R. China

^2^Key Laboratory of Biotechnology and Bioresources Utilization, Dalian Minzu University, Dalian 116650, P. R. China

*Corresponding author.

E-mail : [jydai@dlut.edu.cn](mailto:jydai@dlut.edu.cn) (J.Y. Dai)

**1. GC analysis of the aldol condensation reaction of 5-MF and acetoin**

**Fig. S1** GC analysis of the aldol condensation reaction of acetoin and 5-MF. t_R_ 6.653 min, 5-MF; t_R_ 13.506 min, Product I; t_R_ 15.562 min, Product II; t_R_ 16.555 min, Product III; t_R_ 17.846 min, Product IV; t_R_ 14.687 min, imine intermediate.

**2. GC-MS analysis for the four C10 products and intermediate**


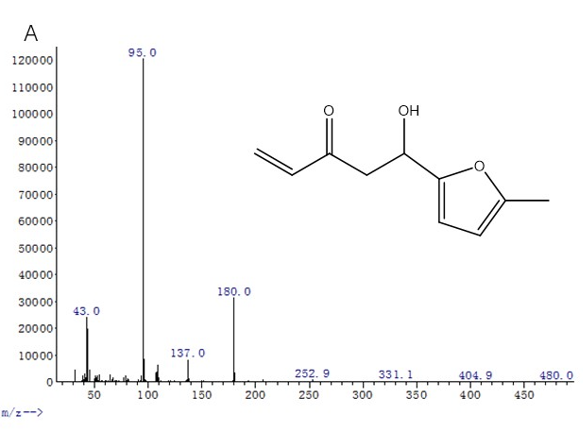


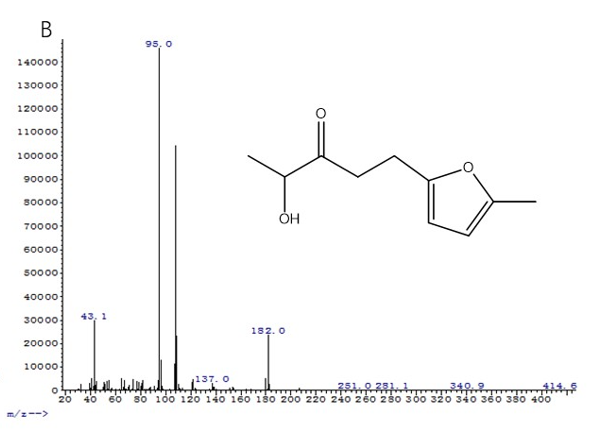


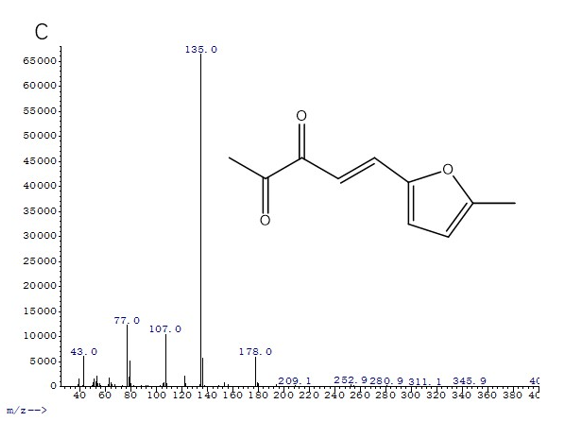


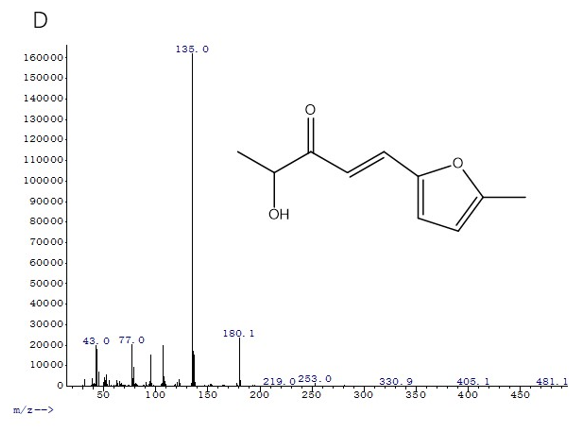


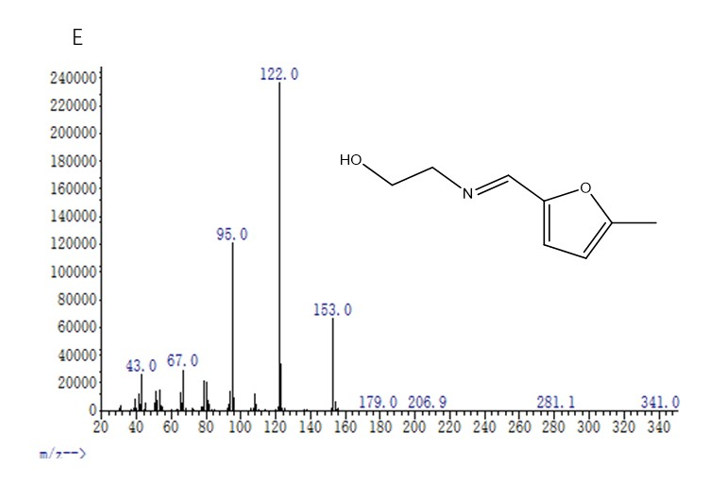


**Fig. S2** GC-MS diagram of the four C10 products and intermediate.

A, Product I (5-hydroxy-5-(5-methylfuran-2-yl)pent-1-en-3-one);

B, Product II (4-hydroxy-1-(5-methylfuran-2-yl)pentan-3-one);

C, Product III (5-(5-methylfuran-2-yl)pent-4-ene-2,3-dione);

D, Product IV (4-hydroxy-1-(5-methylfuran-2-yl)pent-1-en-3-one);

E, Intermediate (2-(((5-methylfuran-2-yl)methylene)amino)ethan-1-ol).

**3. ^1^H NMR spectra of product Ⅳ and intermediate**


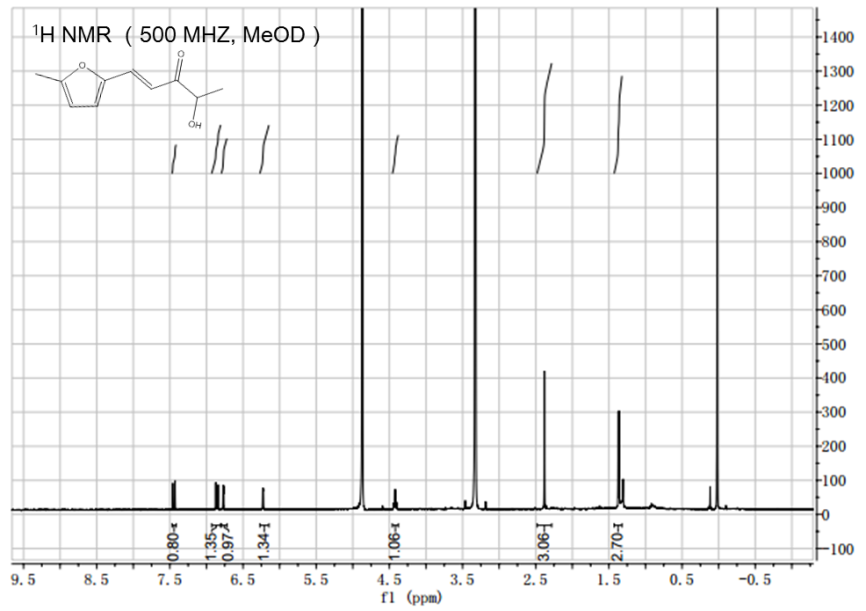


**Fig. S3** ^1^H NMR spectra of product Ⅳ synthesized from 5-MF and AC.

^1^H NMR (500 MHZ, CDCl_3_)

**Fig. S4** ^1^H NMR spectra of intermediate.
